# Medial Plantar Artery Perforator Flap Based on an Aberrant Peroneal Artery Branch for Lower Limb Reconstruction: A Case Report

**DOI:** 10.7759/cureus.104534

**Published:** 2026-03-02

**Authors:** Bappy Basak, John Gibson, Florin Panduru, Nicholas Marsden, Ahmed Emam

**Affiliations:** 1 Burns and Plastic Surgery, Morriston Hospital, Swansea, GBR

**Keywords:** arteria peronea magna, lower limb reconstruction, medial plantar artery perforator flap, posterior tibial artery hypoplasia, vascular anomaly

## Abstract

The medial plantar artery perforator flap (MPAF) is a fasciocutaneous flap that typically arises from the posterior tibial artery (PTA). It is a reliable reconstructive option in both trauma and elective settings. We present two successful cases of distal lower limb reconstruction using an MPAF arising from the peroneal artery. In both patients, CT angiography demonstrated an absent or hypoplastic PTA with a dominant peroneal artery supplying the plantar foot. Recognition of vascular anomalies, such as peroneal artery dominance, and the use of preoperative imaging are crucial for surgical planning. To our knowledge, these are the first reported cases of MPAFs originating from the peroneal artery.

## Introduction

Reconstruction of soft tissue defects of the distal leg and foot remains challenging due to limited local tissue availability. The posterior tibial artery (PTA) plays a vital role in lower limb reconstruction as a recipient vessel for free tissue transfer or as the vascular pedicle for local flaps. However, anatomical variations, such as PTA hypoplasia or aplasia, can limit reconstructive options by reducing the availability of conventional techniques. Since its first description, the medial plantar artery perforator flap (MPAF) has become a reliable option for soft tissue coverage of the heel and plantar foot. Its use is especially valuable when traditional recipient vessels, such as the PTA, are absent or compromised [1].

The MPA typically arises as a terminal branch of the PTA and divides into superficial and deep branches. Perforators from the medial branch of the deep MPA supply the medial plantar flap, with venous drainage occurring through accompanying venae comitantes and communicating superficial veins. Due to its predictable vascular anatomy and robust tissue quality, it offers a versatile alternative in such cases. While multiple studies have described its use based on the PTA, there is limited data on the use of the MPAF from an aberrant peroneal artery [2,3].

Here, we report two cases of open distal lower limb fractures with absent PTA and a dominant peroneal artery supplying the plantar foot. Both patients underwent successful soft tissue reconstruction using an MPAF raised on a branch of the peroneal artery, demonstrating its effectiveness in anatomically challenging scenarios.

The abstract of this case report was presented as a poster at the British Association of Plastic, Reconstructive and Aesthetic Surgeons Conference, Montego Bay, Jamaica (October 23-26, 2024), and at the World Society for Reconstructive Microsurgery Conference, Barcelona, Spain (April 23-26, 2025).

## Case presentation

Case 1

An 80-year-old female presented to the emergency department following a fall at home. She sustained an isolated Weber B2 right ankle bimalleolar open fracture. Examination revealed a small medial ankle wound with intact distal sensation. The anterior tibial artery was palpable; however, the PTA was not. Her past medical history included hypertension, mild chronic obstructive pulmonary disease, and atrial fibrillation. She was taking apixaban for anticoagulation.

**Figure 1:**
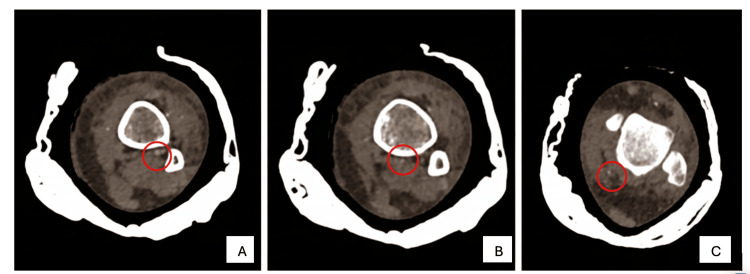
CT scan axial view Axial plane CT angiography from proximal (A) to distal (C) of the right leg showing absence of the PTA and a dominant peroneal artery (circled). PTA, posterior tibial artery

As part of surgical planning, CT angiography was performed, which revealed the absence of the PTA and a dominant peroneal artery. The peroneal artery coursed posterior to the interosseous membrane and medial to the flexor hallucis longus, following the typical PTA trajectory into the sole (Figure 1).

The patient underwent single-stage open reduction and internal fixation with fibular intramedullary nailing, syndesmotic screw fixation, medial malleolus repair, and soft tissue coverage using an MPAF. The flap was based on the terminal branch of the aberrant peroneal artery, and the donor site was covered with a split-thickness skin graft. Postoperative recovery was uneventful, and at six-week follow-up, both the flap and fixation showed satisfactory results.

Case 2

A 45-year-old female, previously healthy, sustained an open comminuted distal tibia and fibula fracture following a fall. On examination, she had significant soft tissue damage over the distal medial leg, classified as Gustilo-Anderson grade IIIB after initial debridement. Distal neurovascular examination was normal; however, the PTA was not palpable.

**Figure 2:**
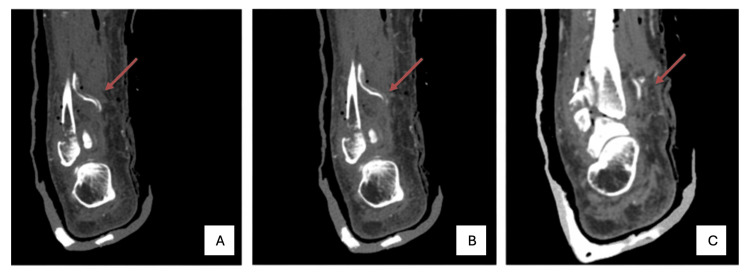
CT scan coronal view Coronal plane CT angiography showing the course of the dominant PTA from lateral (A) to medial (C) at the level of the ankle. PTA, posterior tibial artery

She underwent staged reconstruction. Initial management included wound debridement, application of a vacuum-assisted closure dressing, and temporary external fixation. As part of surgical flap planning, CT angiography revealed a hypoplastic or absent PTA with a dominant peroneal artery supplying the plantar foot (Figure 2).

Definitive fixation was achieved with intramedullary tibial and fibular nailing. Free flap reconstruction was not feasible due to the aberrant arterial anatomy. Soft tissue coverage was accomplished using an MFAP based on a branch of the peroneal artery. The donor site was managed with a dermal regeneration template followed by a split-thickness skin graft.

At over 10 months of follow-up, the flap remained viable with no complications.

## Discussion

Technical considerations

The MPAF is a reliable soft tissue reconstructive option for foot and ankle surgery. The flap-raising technique generally remains the same even in cases of arterial variation, such as peronea magna, where the PTA is small or absent, and the peroneal artery becomes the main blood supply to the foot. Preoperative Doppler ultrasound is used to identify perforators, and flap markings are made from just posterior to the medial malleolus to the first web space or along a sustentaculum tali-first metatarsal axis [2,4,5]. A thigh tourniquet is applied for a bloodless field [2]. Flap elevation begins with an incision near the abductor hallucis, and dissection is carried out in a suprafascial plane [2]. The key perforators, usually around 0.3 mm in diameter, are identified between the abductor hallucis and flexor digitorum brevis muscles [2,4] and are preserved with surrounding tissue to maintain circulation [2].

The MPA is traced proximally after detaching the abductor hallucis origin [4]. Care is taken to preserve both the artery and nerve branches to prevent functional loss, particularly sensation to the medial great toe [4]. The flap is islanded by ligating the artery distally and isolating a neurovascular pedicle of approximately 3 cm [5]. Flap viability is confirmed after tourniquet release. Primary closure of the donor site is possible if the flap is ≤2 cm; otherwise, an immediate split-thickness skin graft or delayed graft with a biodegradable temporizing matrix may be used. Venous drainage is enhanced by preserving superficial veins connecting to the saphenous system, which is vital given the small caliber of the accompanying deep veins [2,4,5].

Clinical implications

Managing soft tissue defects in distal lower limb injuries remains challenging, particularly in trauma settings where local tissue is limited. Free flaps are often used for distal leg defects, with the PTA commonly serving as the donor artery [6,7]. However, arterial injury or anatomical variations may render conventional options unsafe or unfeasible. Improved understanding of perforator flaps has expanded reconstructive options in such scenarios, including the MPAF, which has been established as a dependable option for heel and medial foot defects due to its well-defined vascular anatomy [8].

Traditionally, the MPAF arises from the MPA, a branch of the PTA. However, vascular variations are not uncommon. In approximately 3.3% of cases, the peroneal artery (peronea magna) plays the dominant role in distal leg perfusion in the absence or hypoplasia of the PTA [9]. In our unit, this variation was identified in seven of 420 cases (approximately 2%) over the last five years. In such scenarios, conventional reconstructive options may be unsafe or impossible. While previous studies have extensively described the MPAF based on the PTA, to our knowledge, no reports have described its use when the flap is based on an aberrant peroneal artery.

Preoperative CT angiography is critical for surgical planning, allowing identification of vascular variations and mapping of plantar perfusion. In an observational study, CT angiography identified vascular abnormalities in 11% of cases, with peronea magna present in 0.9% of cases [10]. A retrospective study also reported a relationship between abnormal CT angiography findings and flap failure [11]. These findings underscore the importance of routine preoperative vascular imaging in complex distal lower limb reconstructions.

From a functional perspective, the MPAF remains a viable option even in cases of anatomical variation. Both patients in this report had successful flap reconstruction with minimal donor-site morbidity. Harvesting this flap, however, requires meticulous dissection and precise anatomical knowledge. These cases highlight the first reported safe and effective use of an MPAF based on a dominant peroneal artery in the presence of an absent or hypoplastic PTA. Larger studies would be beneficial to further validate the safety and reproducibility of this approach.

## Conclusions

Although the MPAF is a well-established option for distal lower limb reconstruction, these cases demonstrate its feasibility even in the presence of anatomical variations such as peronea magna. Successful use of the flap in patients with absent or hypoplastic posterior tibial arteries highlights its versatility and reliability in anatomically challenging scenarios.
